# Detecting cervical esophagus with ultrasound on healthy voluntaries: learning curve

**DOI:** 10.1186/s13089-023-00315-8

**Published:** 2023-05-01

**Authors:** Paul-Georges Reuter, Chris Ballouz, Thomas Loeb, Tomislav Petrovic, Frédéric Lapostolle

**Affiliations:** 1 Service des Urgences, SAMU, SMUR, CHU Pontchaillou, Université Rennes, Rennes, France; 2 Équipe Soins Primaires et Prévention, Université Paris-Saclay, UVSQ, Univ. Paris-Sud, Inserm, CESP, 94807 Villejuif, France; 3 Samu des Hauts-de-Seine, Assistance Publique-Hôpitaux de Paris, Hôpital Raymond Poincaré, 92380 Garches, France; 4 SAMU 93 - UF Recherche-Enseignement-Qualité, Université Paris 13, Sorbonne Paris Cité, Inserm U942, Hôpital Avicenne, Assistance Publique-Hôpitaux de Paris, 125, Rue de Stalingrad, 93009 Bobigny, France

**Keywords:** Tracheal ultrasound, Endotracheal tube placement, Education, Simulation, Learning curve

## Abstract

**Background:**

The objective of this study was to determine the learning curve of tracheal−esophageal ultrasound by prehospital medical and paramedical staff.

**Methods:**

A single-center prospective study was carried out at a French EMS (SAMU 92). Volunteer participants first received a short theoretical training through e-learning, followed by two separate hands-on workshops on healthy volunteers, spaced one to two months apart. Learners were timed to obtain the tracheal–esophageal ultrasound target image 10 consecutive times. The first workshop was intended to perform a learning curve, and the second was to assess unlearning. The secondary objectives were to compare performance by profession and by previous ultrasound experience.

**Results:**

We included 32 participants with a mean age of 38 (± 10) years, consisting of 56% men. During the first workshop, the target image acquisition time was 20.4 [IQR: 10.6;41] seconds on the first try and 5.02 [3.72;7.5] seconds on the 10th (*p* < 0.0001). The image acquisition time during the second workshop was shorter compared to the first one (*p* = 0.016). In subgroup analyses, we found no significant difference between physicians and nurses (*p* = 0.055 at the first workshop and *p* = 0.164 at the second) or according to previous ultrasound experience (*p* = 0.054 at the first workshop and *p* = 0.176), counter to multivariate analysis (*p* = 0.02).

**Conclusions:**

A short web-based learning completed by a hands-on workshop made it possible to obtain the ultrasound image in less than 10 s, regardless of the profession or previous experience in ultrasound.

## Background

Upper airway management is often a critical step in the management of critical patients. The main causes of emergency recourse to orotracheal intubation are cardiac arrest (44%), neurological distress (16%), and toxic coma (15%) [1]. In prehospital settings, the rate of esophageal intubation is as high as 30% after the first attempt [2, 3], increasing the risk of gastric distension and regurgitation [4, 5]. If not quickly identified, the main complication is hypoxia potentially leading to cardiac arrest (CA) and to the patient’s death, or at least inducing severe neurological sequelae [6].

To check the endotracheal position of the intubation tube, it is currently recommended to combine several methods: direct laryngoscopy, pulmonary and gastric auscultation, compliance and filling of the ventilation bag, visualization of mist inside the tube, all necessarily associated with capnography. None of these methods used separately can confirm the intratracheal position of the tube with a 100% positive or negative predictive value [7]. The evaluation of the performance of these different methods in emergency medicine showed a sensitivity of 93% and a specificity of 97% for capnography and a sensitivity of 94% and a specificity of 83% for auscultation, all patients combined [8, 9]. In the prehospital setting, capnography, combined with auscultation, is currently considered the gold standard for checking the position of the intubation tube, according to the recommendations of the European Resuscitation Council Guidelines of 2015 [10]. However, it is limited by its low sensitivity in the context of CA (64%), and by the need to perform 5 to 10 breath cycles before being able to interpret the curve.

Tracheal–esophageal ultrasound can identify esophageal or tracheal intubation statically (after intubation was performed) or dynamically (during intubation, with the help of a second operator). Thus, the technique showed a sensitivity of 98% and a specificity of 96% for all patients combined, including those undergoing CA [11]. In another study, ultrasound confirmed the position of the intubation tube in 36 s, compared to 52 s with auscultation and 62 s with capnography [12].

On the ultrasound image, the esophagus appears mostly in the shape of a rosette, its lumen normally completely collapsed and therefore invisible. In the case of esophageal intubation, the tube is distending the lumen making it "visible" by the air it contains, showing the “doubled trachea” sign. This sign confirms esophageal intubation with a sensitivity of 86 to 100%, even in the case of CA [11, 12]. The absence of this sign confirms endotracheal intubation.

Early identification of esophageal intubation could reduce the associated morbidity by reducing the time for reintubation. Most of the studies evaluating the performance of ultrasound in this indication have been carried out in hospital settings. In a study carried out on cadavers, a short training for “prehospital healthcare providers” improved the identification of esophageal intubation by ultrasound with a sensitivity increasing from 55 to 96% [13]. The objective of our study was to establish the learning curve for the correct performance of tracheal−esophageal ultrasound by prehospital staff on healthy volunteers.

## Methods

### Study design and setting

Our work was a monocentric descriptive prospective study carried out from March to June 2021 by the French Emergency Medical Service of department 92 [Service d’Aide Médicale Urgente (SAMU) 92] team of Raymond Poincaré University Hospital, composed of 60 doctors and 30 nurses or nurse anesthetists.

The study was approved by a local ethics committee. An IRB-type declaration has been registered under the number 2022-A01184-39. Participation in the study was voluntary. No material or financial compensation was foreseen. The database has been declared to the National Commission for Computing and Liberties under the number 2227441.

#### Selection of participants

The population studied was that of the SAMU 92 teams, namely medical staff (seniors, residents, medical students) and nurses or nurse anesthetists, regardless of their experience and prior practice of ultrasound.

The criterion for non-inclusion was the regular use, over the past three months, of ultrasound in clinical practice for esophageal visualization.

#### Protocol

The participants first received theoretical training, then two hands-on workshops on healthy volunteers (models), spaced 1 to 2 months apart.

The theoretical training was provided in e-learning, available on the SAMU 92 website. The didactic presentation, in the form of a commented slide show including iconography and videography, was to be consulted before accessing hands-on workshops. Participants could consult the presentation as many times as necessary. It contained (i) the basics of ultrasonography: basic physics on ultrasound and beam formation, use of the equipment, transverse and longitudinal incidences; (ii) an anatomical reminder of the cervical region as well as its sonographic aspects; (iii) the techniques for tracheal−esophageal ultrasound; and (iv) the ultrasound loops with normal views, swallowing artifacts, tracheal intubation, and esophageal intubation.

A hands-on workshop then brought together one or two study investigators, a group of two to four participants and two models who could be learners in turn. The ultrasound device was composed of an ultraportable high-frequency linear probe (Lumify^®^, Philipps^™^) connected to a tablet computer (SAMSUNG^™^ A5). At the start of each hands-on workshop, one of the investigators recalled the ultrasound technique without demonstrating and then answered all possible questions. The models were placed in the supine position, each on a separate examination table. Each participant was placed between the two models with, at his side, the ultrasound device switched on in soft tissue mode, ready to use. He had to perform 10 tries, changing the model at each attempt.

The ultrasound technique involved several steps: (i) manipulation of the linear probe, (ii) transverse placement of the probe on the anterior cervical midline to visualize the trachea, (iii) sliding down to the jugular notch, and (iv) translating the probe to the left in order to visualize a tracheal−esophageal cross section.

#### Measurements

The investigator was responsible for collecting information from each participant by completing a specific form (Appendix 1). Participant characteristics included: age, gender, function, level of training in ultrasound, and frequency of usage: none, occasional (more than once a month), frequent (more than once a week). The frequency of usage was then split into regular (occasional or frequent practice) or no usage (none).

For each attempt, the acquisition time and the number of attempts to achieve a tracheal−esophageal cross section were recorded. The timer was started by the investigator as soon as the learner was taking the ultrasound probe (T0). When the learner thought he had obtained the tracheal−esophageal section, the timer was paused by the investigator (intermediate time, Ti). The investigator then controlled the ultrasound image and asked the model to swallow down to provoke an air artifact in his esophagus. In case of non-visualization of the artifact, the timer was restarted from Ti and a new attempt was counted. If the air artefact was visualized the test was terminated and Ti was the final acquisition time (final time, Tf).

If the image was still not obtained within 3 min, the investigator stopped the timer at Tf = 180 s. The try was then considered as completed and counted as a failure. A new explanation of the technique to the participant was carried out in another room, this time with a demonstration. The participant was then resuming his session of tries.

During the second hands-on workshop, 1 to 2 months after the first one, the frequency of use of tracheal−esophageal ultrasound since the first session was added to previous data.

#### Outcome

The main objective was to define the learning curve of ultrasound identification of a tracheal−esophageal cross section.

The secondary objectives were to assess the risk of unlearning, i.e., the level of knowledge retention, through time and to compare performance according to the level of ultrasound experience.

#### Analysis

Quantitative variables are expressed as mean and standard deviation or as median and interquartile range depending on the number of data and/or their distribution. They were compared by repeated data tests. Qualitative variables are presented as numbers and percentages. They were compared by chi-square tests, after checking the conditions of use.

The distribution of the image acquisition time not following a Normal law and its comparisons according to the test were carried out by a Friedman test. The effect size of the Friedman test was determined using a Kendall’s W test. Comparisons of image acquisition times as a function of several factors were made using mixed ANOVA type tests. The first kind of risk (alpha) was set at 5%. Analyses were performed using R software (version 3.6.0).

## Results

### Characteristics of participants

We included 32 participants, 18 men and 14 women. The characteristics of the population are presented in Table 1. The average age of the participants was 38 (+ 10) years, with extremes of 21 and 61 years. 24 (75%) participants were physicians and 8 (25%) were nurses.

**Table 1:** Characteristics of the 32 learners

Sex ratio (M/F)	1.29
Age	38 (± 9.8)
Fonction	
Senior doctors	11 (34%)
Junior doctors	7 (22%)
Interns	6 (19%)
Nurses	5 (16%)
Nurse anesthetists	3 (10%)
Previous ultrasound training^a^	
Specific specialty course (emergency medicine internship)	13 (40%)
Locally provided training	9 (28%)
Postgraduate university course	6 (19%)
Postgraduate private course	3 (9%)
None	6 (19%)
Use of ultrasound in clinical practice:	
Frequently	13 (41%)
Occasionally	10 (31%)
Never	9 (28%)

Quantitative variables are presented as mean values with standard deviation. The qualitative variables are presented as numbers and percentages

^a^Several trainings are possible per individual

Six participants had never received any training on ultrasound in emergency medicine. Nineteen (59%) had a university education. Thirteen (41%) participants declared a frequent practice of ultrasound. None of the nurses was practicing ultrasonography.

### Primary outcome: the learning curve

**Fig. 1:**
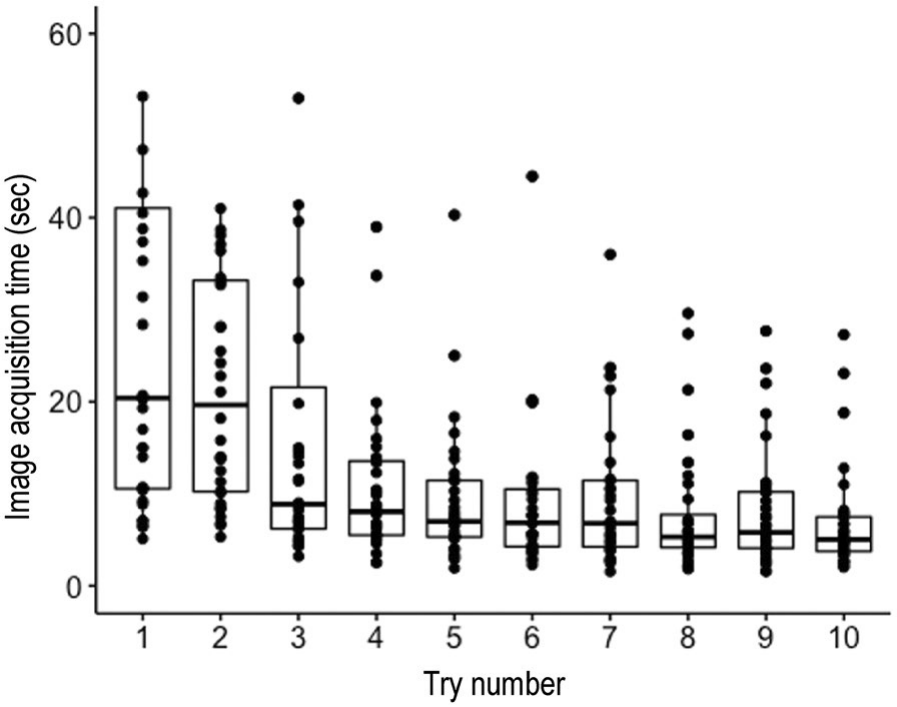
Graphical representation of the learning curve with median and interquartile ranges over all participants (*n* = 32)

The evolution of the image acquisition time during the 10 tries for each learner is presented in Appendices 1 and 2. The distribution of the image acquisition times according to the try is presented in Fig. 1 and Table 2. There was a statistically significant reduction in acquisition times as the tries progressed (Friedman’s test, *p* < 10–4) but with a moderate effect (W Kendall test at 0.44). When comparing times 2 per 2, there was no significant difference between try 1 and 2. However, the time difference was statistically significant between the first and all other tries (Table 2).

**Table 2:** Image acquisition time at the end of the first workshop expressed in seconds and presented in median and interquartile ranges [25%; 75%], and comparison with the acquisition time of the first trial

Try number	Target image acquisition time (in seconds)	p^a^
1	20.4 [10.6;41]	–
2	19.6 [10.2;33.2]	1
3	8.85 [6.2;21.6]	0.048
4	8.05 [5.5;13.5]	< 0.0006
5	7 [5.3;11.4]	< 0.0001
6	6.85 [4.25;10.5]	0.002
7	6.8 [4.22;11.4]	0.0005
8	5.3 [4.15;7.75]	< 0.0001
9	5.8 [4.08;10.2]	0.0004
10	5.02 [3.72;7.5]	< 0.0001

^a^Comparison with the acquisition time of the first trial

### Risk of unlearning

**Fig. 2:**
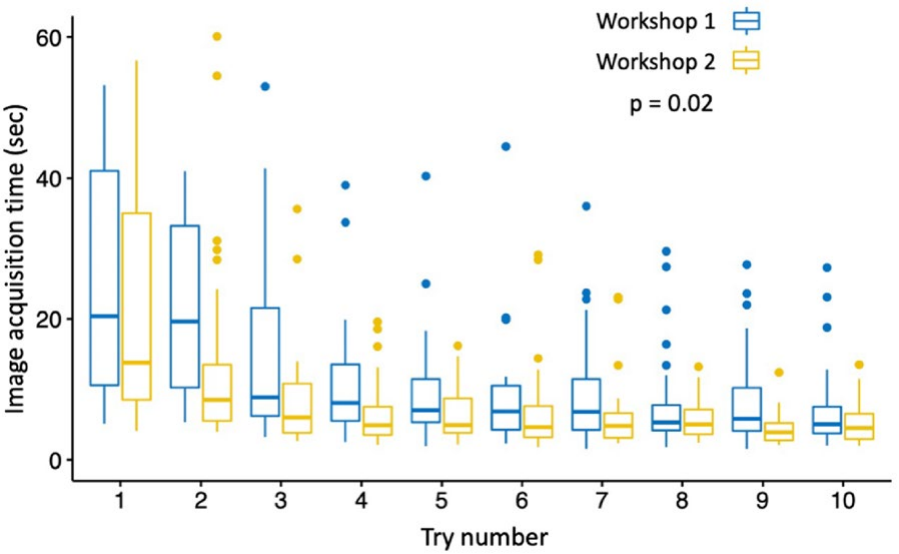
Graphical representation of acquisition times for workshop 1 (learning *n* = 32) and workshop 2 (unlearning, *n* = 29)

Of the 32 participants, 29 (91%) were able to be reassessed within 1 to 2 months after the first hands-on workshop. The distribution of image acquisition times according to the try and according to the workshop is presented in Table 3 and Fig. 2. The acquisition time was significantly lower during the second workshop (*p* = 0.02). There was no interaction between the series and try.

**Table 3:** Comparison of image acquisition times for workshops 1 and 2 expressed in seconds and presented as median and interquartile ranges [25%; 75%]. The difference was statistically significant (*p* = 0.02)

Try number	Workshop 1 (*n* = 32)	Workshop 2 (*n* = 29)
1	20.4 [10.6;41]	13.8 [8.5;35]
2	19.6 [10.2;33.2]	8.5 [5.5;13.5]
3	8.85 [6.2;21.6]	6 [3.8;10.8]
4	8.05 [5.5;13.5]	4.88 [3.5;7.5]
5	7 [5.3;11.4]	4.9 [3.8;8.7]
6	6.85 [4.25;10.5]	4.6 [3.18;7.6]
7	6.8 [4.22;11.4]	4.8 [3.1;6.6]
8	5.3 [4.15;7.75]	5 [3.6;7.1]
9	5.8 [4.08;10.2]	3.89 [2.76;5.2]
10	5.02 [3.72;7.5]	4.5 [2.93;6.5]

### Impact of experience

**Fig. 3:**
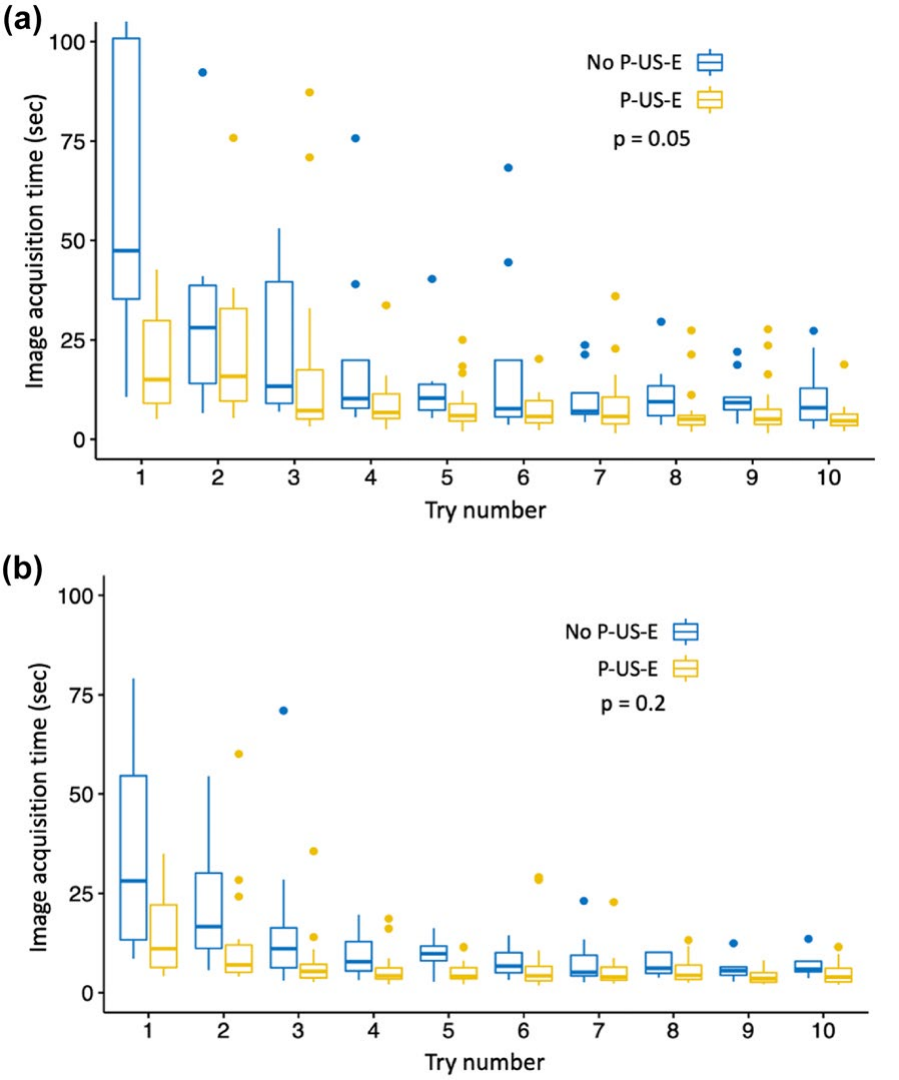
Graphical representation of acquisition times and comparison according to previous ultrasound experience (P-US-E) at the end of workshop 1 (**a**) and workshop 2 (**b**)

For the first workshop, the distribution of image acquisition times according to try did not differ according to ultrasound experience (*p* = 0.05), (Fig. 3a and Table 4). There was no experience−try interaction. For the second workshop, the distribution of image acquisition times according to the try did neither differ according to the experience (*p* = 0.2), (Fig. 3b and Table 5). There was neither experience-try interaction. The distribution of image acquisition times according to the try and to ultrasound experience at three levels is presented in the appendices.

**Table 4:** Comparison of image acquisition times between participants with and without previous ultrasound experience (P-US-E) at the end of the first workshop expressed in seconds and presented in median and interquartile ranges [25%; 75%]

Try number	P-US-E (*n* = 23)	No P-US-E (*n* = 9)
1	15 [9.02;29.9]	47.4 [35.3;101]
2	15.8 [9.59;32.9]	28.1 [14;38.7]
3	7.2 [5.09;17.4]	13.3 [9;39.6]
4	6.7 [5.21;11.4]	10.2 [7.8;19.9]
5	5.9 [4.58 8.9]	10.3 [7.3;13.8]
6	5.7 [4.1;9.7]	7.7 [5.6;19.9]
7	5.7 [3.84;10.6]	7 [6.3;11.6]
8	5 [3.6;5.95]	9.4 [5.9;13.4]
9	5 [3.72;7.5]	9.2 [7.4;10.5]
10	4.6 [3.45;6.3]	7.9 [4.8;12.8]

The difference was not statistically significant (*p* = 0.05)

**Table 5:** Comparison of image acquisition times between participants with and without previous ultrasound experience (P-US-E) at the end of the second workshop expressed in seconds and presented in median and interquartile ranges [25%; 75%]

Try number	P-US-E (*n* = 21)	No P-US-E (*n* = 8)
1	11 [6.3;22.1]	28.2 [13.3;54.6]
2	6.94 [5.1;12]	16.6 [11.1;30.1]
3	5.3 [3.7;7.1]	11 [6.23;16.3]
4	4.2 [3.45;6.2]	7.75 [5.42;12.8]
5	4.1 [3.5;6.25]	9.75 [7.98;11.7]
6	4.2 [2.96;6.6]	6.65 [4.97;10.0]
7	3.9 [3.1;6.37]	5.1 [4.16;9.35]
8	4.3 [3.33;6.9]	6.1 [4.85;10.1]
9	3.5 [2.6;5]	5.5 [4.36;6.4]
10	3.9 [2.66;6.1]	5.86 [5.25;7.85]

The difference was not statistically significant (*p* = 0.2)

### Multivariate analysis

The “experience”, “trial” and “try” were variables retained to analyze the factors influencing the image acquisition time. The results are presented in Table 6. All the variables had an effect on the acquisition time. We found no inter-variable interaction.

**Table 6:** Multivariate analysis of variables having an impact on image acquisition times

Variable	F	p
Previous ultrasound experience (P-US-E)	5.657	0.021
Workshop	6.376	0.014
Try	16.458	< 0.0001
P-US-E:Workshop	0.829	0.37
P-US-E:Try	1.030	0.36
Workshop:Try	0.650	0.52
P-US-E:Workshop:try	1.550	0.22

## Discussion

After a theoretical online training and a first hands-on workshop, participants identified the tracheal−esophageal cross section in less than ten seconds, a time well below the 36.5 s previously reported by Chowdhury et al. SPS:refid::bib12(12). This deviation could be mostly explained by the inclusion of intubation time in their analysis but also by the population studied. Indeed, the ultrasound experience clearly influenced the acquisition time in our study in multivariate analysis. Chowdhury et al. enrolled untrained first-year residents, whereas three-quarters of our participants were trained in ultrasound. However, their image acquisition times were similar at the end of both workshops, regardless of ultrasound experience. In our population, no nurse had previously been trained in ultrasound. Thus, our results support the use of tracheal−esophageal ultrasound by nurses. Among North American paramedics, the esophageal identification rate improved after a short training period with sensitivity increasing from 55 to 96% [13]. In another trial, a ten-minute online tutorial was evaluated by physicians. The correct position of the airway tube was detected on video loops with a sensitivity of 98% and a specificity of 100% [14].

The target image acquisition time has rapidly stabilized below ten seconds from the 3rd try. This skill was maintained over time. When reassessed at one or two months, the image acquisition times were shorter. Performance has improved with every new attempt and remained satisfying between the first and second workshop. There was therefore a good knowledge retention, but, however, with a need for a refresher.

Tracheal−esophageal ultrasound could secure the management of the airway in a prehospital setting. In patients in cardiac arrest (CA), more than 30% of first-attempt failures of orotracheal intubation are due to esophageal intubation (*data under submission*). In this situation, capnography requiring several breath cycles before being interpretable has limited performance due to low sensitivity. As for it, the performance of ultrasound to verify the correct positioning of the intubation tube had a sensitivity of 99% and a specificity of 84% for patients in CA [11]. Furthermore, another study showed that the size of the intubation tube (6.0 to 8.0 mm) had no impact on the diagnostic accuracy of ultrasound [15].

All those characteristics make ultrasound a tool of choice in the management of orotracheal intubation in adults. It was even proposed as a first-intension tool for monitoring the location of the intubation tube, being easier and faster to perform, and more accurate than conventional methods [12, 13]. Moreover, this technique is mentioned in the Advanced Cardiac Life Support section of the American Heart Association, subject to a trained practitioner [16]. Finally, the tracheal−esophageal ultrasound could also make it possible to secure the placement of the nasogastric tube after intubation. The performance of ultrasound in this indication has proved to be superior to that of the syringe test [17–21].

The results of our study should be considered with certain limitations. The first is concerning the difficult generalization of our results to other services or populations due to the monocentric nature and the small number of participants, in particular nurses. Our study was based on volunteers, and the nurses' availability was dependent on the operational activity of our EMS. The second is related to the material tested (Lumify® linear probe, connected to a tablet computer). The transposition of our results to other devices is debatable, although a difference in image acquisition between devices is not established. The third is the availability of ultrasound equipment in a prehospital setting. In 2016, only a third of French EMS were equipped with an ultrasound device [22]. As the rate of ultrasound equipment is gradually increasing, this limit remains relative. The fourth limitation is the simulation nature of the study carried out on healthy volunteers. Thus, we can only hypothesize that these acquired skills could be transposed to clinical practice on patients, in an emergency situation. Furthermore, those patients require additional skill sets, i.e., identification of special artifacts and double-lumen sign. The fifth is that we assessed the ability to acquire a target image and not to detect esophageal intubation. The last limitation lies in the methodology used. A participant alternated between two healthy volunteers. The results might differ if the test had been performed on 10 different volunteers. Learning about the same person is to be feared. Finally, participants were allowed to perform an ultrasound and revise the e-learning training materials after the first hands-on workshop. This could affect skill retention and thus our result in the second workshop.

## Conclusions

This study on the learning curve of tracheal−esophageal ultrasound by a prehospital staff shows encouraging results. Participants little or not accustomed to the practice of ultrasound succeeded after a short training period in acquiring the target image with ultrasound. The acquisition of the image in less than ten seconds makes it a tool of choice for prehospital practice, constrained by operation in a small team. Thus, the skills acquired by both medical and nurse staff could improve the safety of airway management in prehospital settings. However, these conclusions need to be confirmed by further larger studies ruled in real clinical situations.

## Transparency declaration

The lead authors (the manuscript guarantors) affirm that the manuscript is an honest, accurate, and transparent account of the study being reported; that no important aspects of the study have been omitted; and that any discrepancies from the study as planned (and, if relevant, registered) have been explained.

## Data Availability

The statistical code and technical processes are available from the time of publication. Appropriate institutional agreements will be required for anonymized participant data transfer. Requests should be made via email to the corresponding author along with an analysis proposal.

## Image acquisition time according to the level of previous ultrasound experience for the two workshops combined, expressed in seconds and presented in median and interquartile ranges [25%;75%]

**Table:**

Try number	Use of ultrasound in clinical practice		
	Never (*n* = 9)	Occasionally (*n* = 10)	Frequently (*n* = 13)
1	41,8 [14,5;56,7]	17,2 [9,17;33,2]	11 [7,1;20,3]
2	25,5 [12,7;31,1]	13,8 [10,4;28,4]	8,4 [5,5;13,9]
3	11,6 [7,5;28,5]	7,2 [5,07;14,8]	6,2 [4,3;8,3]
4	9,9 [6,4;18]	6,7 [3,88;10,6]	5,1 [4;6,3]
5	10,3 [7,3;13,8]	5,35 [3,98;7,56]	5,1 [3,5;7,7]
6	7,7 [5,3;12,8]	5,33 [3,32;8,5]	5,2 [3,4;8,4]
7	6,8 [4,8;11,6]	6,37 [2,60;14,8]	4,8 [3,48;6,6]
8	7,1 [5;10,1]	4,5 [2,68;6,55]	5,1 [3,5;6,4]
9	6,4 [4,9;10,1]	4 [3,03;6,75]	4,6 [2,76;5,7]
10	6,3 [5,1;11]	4,6 [3,02:6,6]	4,2 [2,93;5,6]

## References

[1] Combes X, Jabre P, Jbeili C, Leroux B, Bastuji-Garin S, Margenet A (2006). Prehospital standardization of medical airway management: incidence and risk factors of difficult airway. Acad Emerg Med Off J Soc Acad Emerg Med.

[2] Galinski M, Wrobel M, Boyer R, Reuter PG, Ruscev M, Debaty G (2023). Risk factors for failed first intubation attempt in an out-of-hospital setting: a multicenter prospective study. Intern Emerg Med.

[3] Le Bastard Q, Pès P, Leroux P, Penverne Y, Jenvrin J, Montassier E (2023). Factors associated with tracheal intubation-related complications in the prehospital setting: a prospective multicentric cohort study. Eur J Emerg Med Off J Eur Soc Emerg Med.

[4] Takeda T, Tanigawa K, Tanaka H, Hayashi Y, Goto E, Tanaka K (2003). The assessment of three methods to verify tracheal tube placement in the emergency setting. Resuscitation.

[5] MacLeod BA, Heller MB, Gerard J, Yealy DM, Menegazzi JJ (1991). Verification of endotracheal tube placement with colorimetric end-tidal CO2 detection. Ann Emerg Med.

[6] Clyburn P, Rosen M (1994). Accidental oesophageal intubation. Br J Anaesth.

[7] Donald MJ, Paterson B (2006). End tidal carbon dioxide monitoring in prehospital and retrieval medicine: a review. Emerg Med J EMJ.

[8] Grmec S (2002). Comparison of three different methods to confirm tracheal tube placement in emergency intubation. Intensive Care Med.

[9] Li J (2001). Capnography alone is imperfect for endotracheal tube placement confirmation during emergency intubation. J Emerg Med.

[10] Soar J, Nolan JP, Böttiger BW, Perkins GD, Lott C, Carli P (2015). European resuscitation council guidelines for resuscitation 2015: section 3. Adult advanced life support. Resuscitation.

[11] Sahu AK, Bhoi S, Aggarwal P, Mathew R, Nayer J, Amrithanand VT (2020). Endotracheal tube placement confirmation by ultrasonography: a systematic review and meta-analysis of more than 2500 patients. J Emerg Med.

[12] Chowdhury AR, Punj J, Pandey R, Darlong V, Sinha R, Bhoi D (2020). Ultrasound is a reliable and faster tool for confirmation of endotracheal intubation compared to chest auscultation and capnography when performed by novice anaesthesia residents—a prospective controlled clinical trial. Saudi J Anaesth.

[13] Kaminski A, Dike NO, Bachista K, Boniface M, Dove C, simon LV (2020). differences between esophageal and tracheal intubation ultrasound view proficiency: an educational study of novice prehospital providers. Cureus.

[14] Chenkin J, McCartney CJL, Jelic T, Romano M, Heslop C, Bandiera G (2015). Defining the learning curve of point-of-care ultrasound for confirming endotracheal tube placement by emergency physicians. Crit Ultrasound J.

[15] Gottlieb M, Holladay D, Nakitende D, Hexom B, Patel U, Serici A (2019). Variation in the accuracy of ultrasound for the detection of intubation by endotracheal tube size. Am J Emerg Med.

[16] Link MS, Berkow LC, Kudenchuk PJ, Halperin HR, Hess EP, Moitra VK (2015). Part 7: adult advanced cardiovascular life support: 2015 American Heart Association Guidelines Update for Cardiopulmonary Resuscitation and Emergency Cardiovascular Care. Circulation.

[17] Brun PM, Chenaitia H, Bessereau J, Leyral J, Barberis C, Pradel-Thierry AL (2012). Ultrasound evaluation of the nasogastric tube position in prehospital. Ann Fr Anesth Reanim.

[18] Brun PM, Chenaitia H, Lablanche C, Pradel AL, Deniel C, Bessereau J (2014). 2-point ultrasonography to confirm correct position of the gastric tube in prehospital setting. Mil Med.

[19] Chenaitia H, Brun PM, Querellou E, Leyral J, Bessereau J, Aimé C (2012). Ultrasound to confirm gastric tube placement in prehospital management. Resuscitation.

[20] Lin T, Gifford W, Lan Y, Qin X, Liu X, Wang J (2017). Diagnostic accuracy of ultrasonography for detecting nasogastric tube (NGT) placement in adults: a systematic review and meta analysis. Int J Nurs Stud.

[21] Mak MY, Tam G (2020). Ultrasonography for nasogastric tube placement verification: an additional reference. Br J Community Nurs.

[22] Bobbia X, Abou-Badra M, Hansel N, Pes P, Petrovic T, Claret PG (2018). Changes in the availability of bedside ultrasound practice in emergency rooms and prehospital settings in France. Anaesth Crit Care Pain Med.

